# Value recognition

**Published:** 2015

**Authors:** VL Purcarea

**Affiliations:** * “Carol Davila” University of Medicine and Pharmacy, Bucharest, Romania

In the economy of knowledge, ingenuity and dynamism, innovation and technology are considered essential levers, and knowledge and creativity key factors. 

Globalization and liberalization have generated a new dynamic of competition, a dynamic competitiveness, in which the focus is on the ability of performing, including in related fields. 

Today we are witnessing a moment when a strong emphasis is placed on the development of some new theoretical models in communication and on the development of innovative methods of observing and measuring the communication behavior. The social interaction, interpersonal communication, mass communication, intercultural communication, research of physical and chemical mechanisms of brain activity, observing in real time some processes which take place at the cellular and molecular level, interest for research and knowledge of the element which offers significance to the human being, the brain, etc., elements which seem to be more often under the “magnifier” of progresses in understanding the human symbolical processes. 

That is why Neurosciences have shaped as a distinct field. The dynamics of neuroscientific research had an exponential development and the social impact is considered very powerful. 

*“However, what makes a brand strong is the collective nature of the perceptions about it” – affirmed the Marketing specialists*. In addition, the perceptions regarding an incontestable personality in the field of research in neurosciences and the echo of its performing and high quality research activity impressed the target public. So, this kind of recognition took place on the occasion of the exceptional medical scientific and educational event organized by the **Foundation of the Society for the Study of Neuroprotection and Neuroplasticity** (SSNN), together with the **Romanian Society of Neurology** (RSN) and **“Iuliu Haţieganu” University of Medicine and Pharmacy** in Cluj-Napoca, under the aegis of the **European Federation of NeuroRehabilitation Societies** (EFNRS) and the ** World Federation for NeuroRehabilitation** (WFNR), - **RoNeuro Brain Days**, which hosted the 5th Edition of the **European Teaching Course on Neurorehabilitation** which took place in the period 1-4 June 2015, in Cluj-Napoca, in “Gheorghe Marinescu” Amphitheater of “Iuliu Haţieganu” University of Medicine and Pharmacy, and, which managed to reunite under one single roof important personalities in the medical field and world scientific research from countries such as Germany, USA, Austria, Italy, United Kingdom, Ukraine and Romania. 

**Fig. 1:**
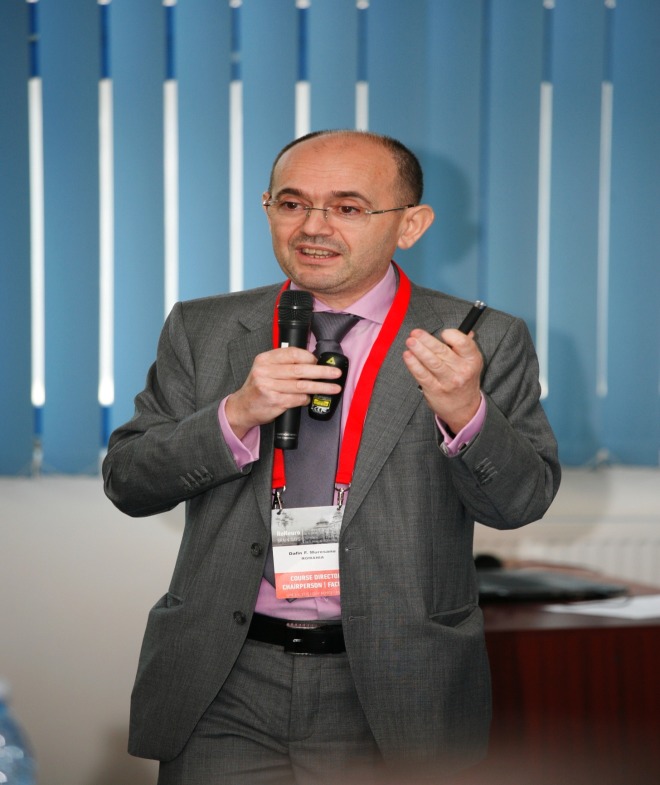
Prof. Dafin F. Mureşanu, MD, President of the Romanian Society of Neurology (RSN) and President of the Society for the Study of Neuroprotection and Neuroplasticity (SSNN)

On this occasion, distinguished **Prof. Michael Chopp, PhD**, was awarded **“Doctor Honoris Causa”** title at the proposal of **Prof. Dafin F. Mureşanu, MD**, President of the Romanian Society of Neurology (SNR) and President of the Society for the Study of Neuroprotection and Neuroplasticity (SSNN), “Iuliu Haţieganu” University of Medicine and Pharmacy in Cluj-Napoca. 

**Fig. 2:**
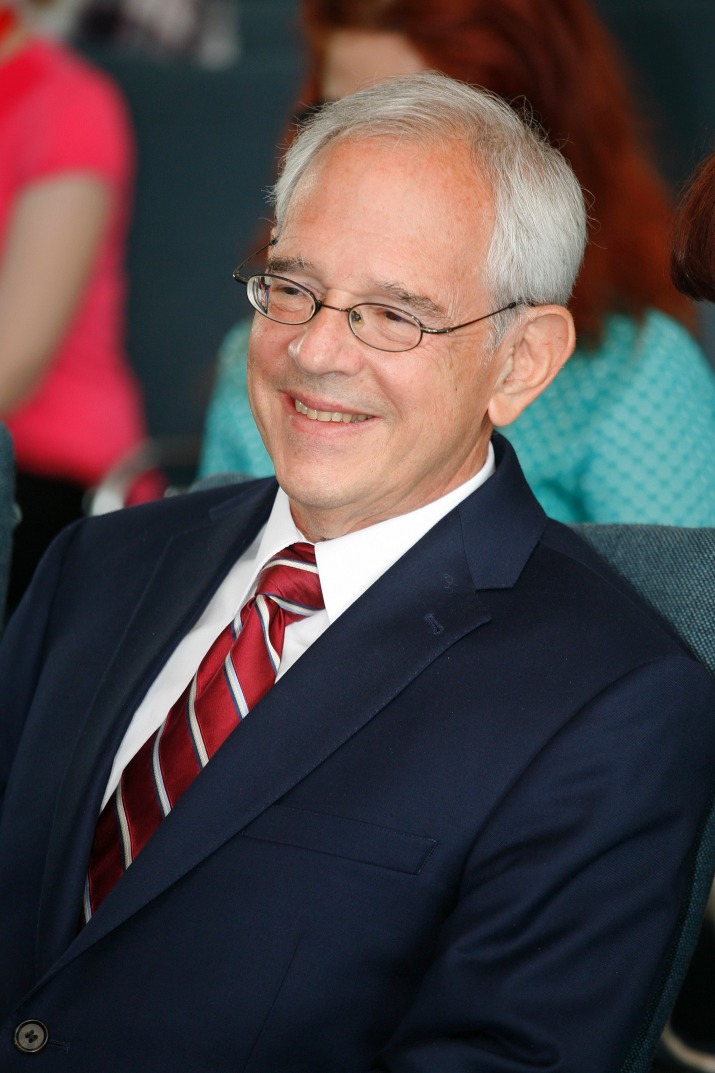
Prof. Michael Chopp, PhD

Michael Chopp, PhD, joined the Henry Ford Health System in Detroit in 1983. He was appointed Vice Chairman for Research of the Department of Neurology in 1991, Scientific Director of the Henry Ford Neuroscience Institute in 1999, and is the Zoltan J. Kovacs Chair in Neuroscience Research. Dr. Chopp is also Distinguished Professor of Physics at Oakland University in Rochester, MI.

**Fig. 3:**
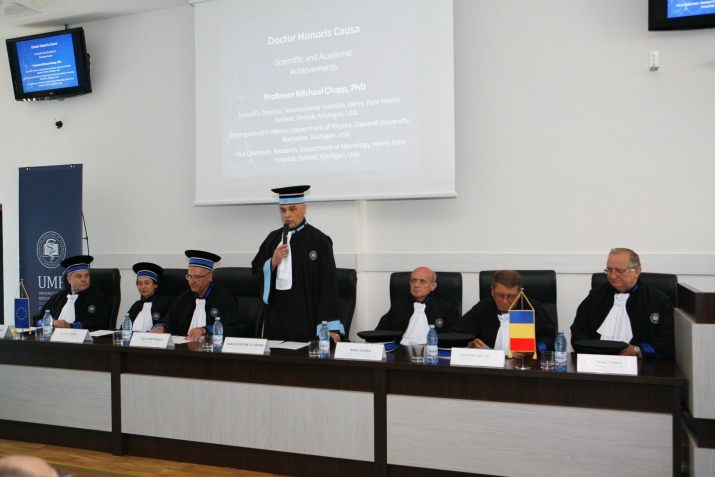
Doctor Honoris Causa Festivity. Members of the presidium

He received his MS and doctorate degrees in Mathematical and Solid State Physics from New York University. After nearly 10 years of working as a Physicist and as a Professor of Physics, Dr. Chopp made a career change and turned his interest to translational research in neuroscience.

Dr. Chopp’s research has primarily focused on: 1) cellular and molecular biology of ischemic cell injury, 2) the pathophysiology of stroke, traumatic brain injury, peripheral neuropathy, multiple sclerosis, and glioma, 3) combination thrombolytic and neuro and vascular protective therapies for stroke, 4) mechanisms of neuroprotection, 5) cell-based and pharmacological neuro-restorative therapies for stroke, traumatic brain injury and neurodegenerative disease, 6) molecular and cellular mechanisms underlying neurogenesis and angiogenesis and the induction of brain plasticity leading to functional and behavioral recovery after neural injury, 7) treatment of glioma, 8) exosomes/ microRNA for the treatment of neurological injury and disease, and 9) magnetic resonance imaging.

**Fig. 4:**
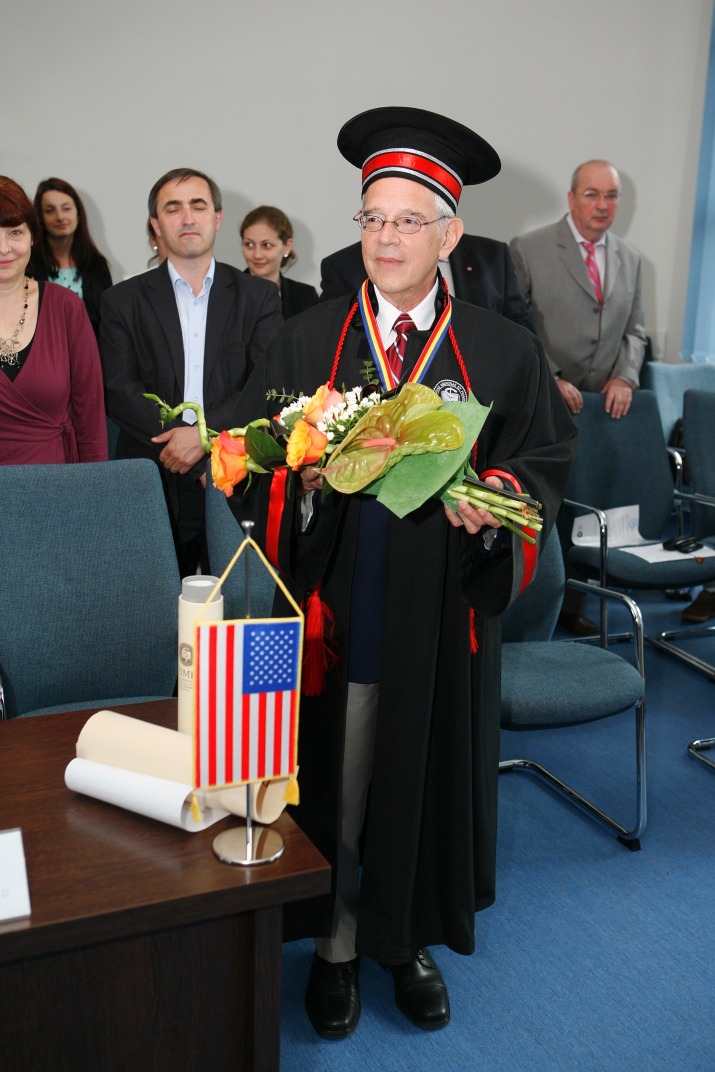
Prof. Michael Chopp, PhD after being awarded the Doctor Honoris Causa title

Dr. Chopp has received multiple awards and recognitions for his research efforts, including the American Heart Association Thomas Willis Lecture Award, the Abraham White Distinguished Science Award, and the Lecture of Excellence and World Stroke Organization Award. 

Dr. Chopp has 623 peer-reviewed publications and has given 414 plenary lectures and invited presentations. He has served on and chaired National Institutes of Health (NIH) study sections and has served as a consultant to government agencies, the U.S. National Institutes of Health, and the pharmaceutical industry. 

A distinctive feature of **Prof. Michael Chopp, PhD** is first his great curiosity, knowledge assimilation through a profound understanding of the facts and data, which at first, were filtrated by his reasoning. What is very impressive is his power of perception, organizing skills and knowledge deepening, as well as the presentation of the most abstract problems in simple and easy to understand forms, especially the high quality and performance of his research activities. 

**Executive Editor** **Assoc. Prof. Dr. Eng. Victor Lorin Purcarea**

